# BSA- and Elastin-Coated GO, but Not Collagen-Coated GO, Enhance the Biological Performance of Alginate Hydrogels

**DOI:** 10.3390/pharmaceutics12060543

**Published:** 2020-06-11

**Authors:** Ahmed Raslan, Laura Saenz del Burgo, Albert Espona-Noguera, Ana María Ochoa de Retana, María Luisa Sanjuán, Alberto Cañibano-Hernández, Patricia Gálvez-Martín, Jesús Ciriza, Jose Luis Pedraz

**Affiliations:** 1 NanoBioCel Group, Laboratory of Pharmacy and Pharmaceutical Technology, Faculty of Pharmacy, University of the Basque Country UPV/EHU, 01006 Vitoria-Gasteiz, Spain; drrayad@gmail.com (A.R.); laura.saenzdelburgo@ehu.eus (L.S.d.B.); albertesponanoguera@gmail.com (A.E.-N.); albertocanibano@gmail.com (A.C.-H.); 2 Biomedical Research Networking Center in Bioengineering, Biomaterials, and Nanomedicine, CIBER-BBN, 28029 Madrid, Spain; 3 Department of Organic Chemistry I, Faculty of Pharmacy and Lascaray Research Center, University of the Basque Country (UPV/EHU), Paseo de la Universidad 7, 01006 Vitoria, Spain; anamaria.ochoaderetana@ehu.eus; 4 Instituto de Ciencia de Materiales de Aragón (Universidad de Zaragoza-CSIC), Facultad de Ciencias, 50009 Zaragoza, Spain; sanjuan@unizar.es; 5 R&D Human Health, Bioibérica S.A.U., 08029 Barcelona E-, Spain; pgalvez@bioiberica.com

**Keywords:** graphene oxide, bovine serum albumin, type I collagen, elastin, alginate hydrogels, cell viability

## Abstract

The use of embedded cells within alginate matrices is a developing technique with great clinical applications in cell-based therapies. However, one feature that needs additional investigation is the improvement of alginate-cells viability, which could be achieved by integrating other materials with alginate to improve its surface properties. In recent years, the field of nanotechnology has shown the many properties of a huge number of materials. Graphene oxide (GO), for instance, seems to be a good choice for improving alginate cell viability and functionality. We previously observed that GO, coated with fetal bovine serum (FBS) within alginate hydrogels, improves the viability of embedded myoblasts. In the current research, we aim to study several proteins, specifically bovine serum albumin (BSA), type I collagen and elastin, to discern their impact on the previously observed improvement on embedded myoblasts within alginate hydrogels containing GO coated with FBS. Thus, we describe the mechanisms of the formation of BSA, collagen and elastin protein layers on the GO surface, showing a high adsorption by BSA and elastin, and a decreasing GO impedance and capacitance. Moreover, we described a better cell viability and protein release from embedded cells within hydrogels containing protein-coated GO. We conclude that these hybrid hydrogels could provide a step forward in regenerative medicine.

## 1. Introduction

Hydrogels are 3D structures capable of supporting living cells and creating a suitable microenvironment that enables cells to maintain their viability. Hydrogels are characterized by excellent properties, such as having enough porosity to allow for the exchange of nutrients and oxygen inside, while releasing waste products and therapeutic proteins outside. Moreover, hydrogels provide protection to embedded cells from the immune system, avoiding their rejection [1] Alginate is the most popular natural biomaterial used in the performance of the aforementioned hydrogels for tissue engineering, due to its high biocompatibility and ease of performance. Alginate hydrogels have been extensively studied for wound healing, drug delivery, cell-based therapies and tissue engineering applications. Although alginate hydrogels retain a structure similar to the extracellular 3D structure [2,3] this inert polymer is unable to mimic interactions with cells, since it inherently lacks cellular adhesion [4]. Therefore, the modification of the alginate surface in order to create a closer bio-mimic support for embedded cells is desired.

In the last few years, the incorporation of nanotechnology into a huge number of materials has shown the improvement of their properties. Thus, for example, graphene oxide (GO), the most studied graphene derivative, has been incorporated into alginate matrices to provide bio-mimetic support, suggesting that is a suitable candidate for the improvement of alginate surface properties and mechanical strength, partly due to its excellent mechanical properties [5,6,7]. In fact, graphene and its derivates can reinforce the physical characteristics of different materials, such as thermoplastic polyurethane [8], hydroxyapatite (HAp) and β-tricalcium phosphate (β-TCP) [9]. GO has also been shown to be a good candidate in the development of drug delivery systems, gene therapy or in the improvement of contrast substances for diagnostic images [10,11,12,13].

GO is produced by the oxidation and exfoliation of natural graphite powder, using various oxidizing agents in a strong acid medium, this being the traditional synthesis method developed by Hummers and colleagues [14]. It shows unique properties, such as high specific surface area (890 m^2^ g^–1^) [15] and mechanical strength (Young’s modulus of ~1.0 and breaking strength of ~130 GPa) [16]. Moreover, the oxidation procedure from graphene to generate GO, provides the material with a high hydrophilicity. In fact, GO is formed by abundant oxygenated groups, such as hydroxyl and epoxy groups on the basal plane, with slight amounts of carboxyl, carbonyl, phenol, lactone and quinone [13], clearly observed by FT-IR spectroscopy [14,15,16]. These groups facilitate the formation of the stable dispersion of the graphene derivate in aqueous media and other polar solvents [17,18,19], also allowing biochemical and bio-conjugation reactions on its basal plane and edges [20]. These reactions facilitate the functionalization of the GO surface with proteins, antibodies and DNA fragments [21,22], providing a wide number of biological applications [23].

Moreover, GO can adsorb proteins and antibodies, providing them with stability against proteolysis [24,25], resulting in an effective platform for protein delivery [26] or biosensors [27,28]. Depending on GO morphology, hydrophobicity [25] and the type of adsorbed protein [25], physical or chemical adsorption on GO can be involved in the adsorption of those proteins. Physical adsorption includes hydrophobic interaction, Van der Waals forces, electrostatic interactions and hydrogen bonds [25,29,30]. Protein adsorption on the GO surface mostly occurs via hydrophobic–hydrophobic interactions through the sp^2^ hybridization of GO [31], with a high affinity for the hydrophobic carbon lattice from the hydrophobic domain of proteins [32]. Van der Waals forces also play an important role in the adsorption of hydrophobic drugs or nanocomposites [27], while electrostatic interactions are generated at lower pH than 6.0 [28]. Hydrogen bonds are particularly described in the adsorption of gases, such as nitrogen oxides, with the formation of hydrogen bonds OH···O (N), between –OH and nitrogen oxides [33]. Moreover, π–π stacking interactions have been described due to the abundant π electrons on the basal plane of the GO surface [30]. On the other hand, the chemical adsorption of proteins on GO provides stability to the proteins against heat, pH and organic solvents [34]. However, this interaction alters the protein structure, decreasing protein functionality or enzymatic activity [35].

However, current studies regarding cytotoxicity with graphene and its derivates are contradictory [36,37]. While some studies reported that GO has no effects on cell behavior at certain doses [38,39], others demonstrated that GO can induce cellular damage [40,41]. On one hand, some studies reported that GO has no effects on the behavior of cells [38,39], such as the high hemocompatibility of pristine and functionalized graphene, even at high concentrations, with red blood cells, platelets and plasma coagulation pathways, the mediation of the activation of cytokines, [38] or on the lack of cytotoxicity of GO at low doses in A549 cells [42]. On the other hand, other studies have demonstrated that this material could induce cellular damage [40,41], such as mitochondrial toxicity and the cell membrane damage of neuronal PC12 cells in a dose-dependent manner with high cytotoxicity even at low concentrations [43] or the cytotoxicity and oxidative stress detected in BF-2 cells at low GO concentrations after 24 h of incubation [44,45]. However, another study demonstrated that low concentrations of GO (≤20 μg/mL) do not show toxicity on human fibroblast cells, while concentrations over 50 μg/mL decrease cell adhesion, induce cell apoptosis and show carbon material within the lysosomes, mitochondrion, endoplasm and cell nucleus [46]. We have also reported that concentrations between 25 and 50 µg/mL GO improve the viability, metabolic activity and membrane integrity of alginate encapsulated myoblasts [10,47,48]. Nevertheless, we also detected adsorption on the GO surface, precluding the release of the studied therapeutic protein, erythropoietin (EPO). We hypothesized that adsorption could occur probably via electrostatic interactions and the formation of hydrogen bonds with oxygenated groups from GO. In addition, the presence of surface defects on the GO surface could also act as active sites where the EPO molecules would be adsorbed. We were able to block the adsorption of EPO by previously incubating GO platelets with fetal bovine serum (FBS), which also further improved the viability of encapsulated cells. 

Following our previous results, we aimed to discern a protein that could prevent the adsorption by GO, instead of using a complex mixture of unknown proteins, such as FBS, and describe and characterize which processes could be involved in the interaction between the selected proteins and GO platelets. Therefore, we have deeply characterized the interaction with three proteins—bovine serum albumin (BSA), type I collagen and elastin—next studying their biological outcomes for embedded myoblasts within alginate hydrogels in the presence of selected protein-coated GO platelets.

## 2. Materials and Methods

### 2.1. Materials

GO suspension was purchased from Graphenea (San Sebastián, Spain). In order to avoid the formation of aggregates, the suspension was diluted to 250 ug/mL in deionized water and sonicated for 1 h before use. BSA, collagen, calcium sulphate and mannitol were purchased from Sigma Aldrich (St. Louis, MO, USA). Elastin was provided by Bioiberica (Barcelona, Spain). Ultrapure low-viscosity and high guluronic (LVG) sodium alginate was purchased from FMC Biopolymer (Sandvika, Norway). Glacial acetic acid was supplied by Panreac. FBS, L-glutamine, Dulbecco’s phosphate-buffered saline (DPBS) and the antibiotic/antimycotic solution were purchased from Gibco. Trypsin-EDTA was purchased from Life Technologies (Carlsbad, CA, USA).

### 2.2. Characterization of GO-Protein Interactions

GO (250 µg/mL) was dispersed in either a BSA solution (900 µg/mL), collagen solution (315 µg/mL) or elastin solution (900 µg/mL). These mixtures were incubated for 2 h at 37 °C. Then, the samples were centrifuged at 14,000 rpm for 15 min and lyophilized in a lyobeta 15 Telstar. The GO and proteins alone were also analyzed in parallel. All of the samples were analyzed in triplicate. Thus, FT-IR spectroscopy measurements were performed with a BRUKER IFS 66/S Spectrometer (Bruker, Billerica, MA, USA), using 32 scans with a resolution of 4 (cm^−1^) in 4000–400 cm^−1^ regions. The Raman spectrum was acquired using a Confocal Raman Imaging Alpha 300 M (Company WITEC) with a 532 nm laser (1 mW laser power, 50× microscope objective, an exposure time of 50 s, and four accumulations). 

### 2.3. Adsorption Capacity Experiments

#### 2.3.1. Effect of Initial Concentration

In order to study the effect of the initial concentration (C_0_) of selected proteins on the GO adsorption capacity (qe), sequential protein concentrations from 0 to 2 mg/mL were incubated with a GO suspension (250 µg/mL) for 2 h at 37 °C. The resulting GO-protein suspensions were centrifuged at 14,000 rpm for 15 min. Then, the quantification of non-adsorbed protein was determined from the supernatant using a BCA kit (Thermo Fisher, Waltham, MA, USA). Absorbance was read at 562 nm on a M 200 TECAN Microplate reader (TECAN Trading AG, Männedorf, Switzerland). Each experiment was performed in triplicate. The percentages of adsorbed proteins were calculated according to Equation (1) and the adsorption capacity qe (µg/µg) was calculated using Equation (2).

(1) $$\mathrm{Adsorption}\;\mathrm{capability}\;(\%)=\frac{\left(C_{0}-\mathrm{Ce}\right)\times 100}{C_{0}}$$

(2) $$\mathrm{Adsorption}\;\mathrm{capacity}\;\mathrm{qe}=\frac{\left(C_{0}-\mathrm{Ce}\right)\times V}{W}$$

where C_0_ (µg/mL) and C_e_ (µg/mL) are the initial and final protein concentrations, respectively; V is the volume of the samples (0.1 mL) and W is the mass of GO (250 µg). 

#### 2.3.2. Adsorption Isotherms

Langmuir and Freundlich adsorption models were applied to study the adsorption isotherm. The Langmuir equation is expressed as follows in Equations (3) and (4):

C_e_/q_e_ = Ce/q_max_ + 1/(q_max_.K_L_) (3)

R_L_ = 1/(1 + K_L_ × C_0_) (4)

where C_e_ (µg/mL) is the concentration of the adsorbed protein at equilibrium, qe is the adsorption capacity (ug/ug), q_max_ (µg/µg) is the maximum amount of protein absorbed per unit weight of GO, K_L_ (mL/µg) is the Langmuir constant related to the surface affinity for the protein, (C_0_) is the initial protein concentration and R_L_ is the separation factor, which describes the essential characteristics of the Langmuir isotherm.

The Freundlich equation was expressed as follows in Equation (5):

Log q_e_ = log K_F_ + 1/n × log C_e_ (5)

where K_F_ and n are the Freundlich constant and intensity adsorption, respectively. 

### 2.4. Kinetic Study of Protein Adsorption

GO (250 µg/mL) was dispersed in either a BSA solution (900 µg/mL), collagen solution (315 µg/mL) or elastin solution (900 µg/mL). These mixtures were incubated at 37 °C and the samples were centrifuged at 14,000 rpm for 10 min at 12,000 rpm after 0, 5, 10, 20, 30 and 80 min of incubation. The supernatants were collected for the quantification of the non-adsorbed proteins with the BCA kit (Thermo Fisher). The results were analyzed using the pseudo-second-order model in order to clarify the nature of the adsorption phenomenon. This pseudo-second-order model is described by Equation (6) [49]:

t/q_t_ = t/qe + 1/k_2_ × (q_e_)^2^ (6)

where q_e_ (µg/µg) and q_t_ (µg/µg) are the adsorption capacity at equilibrium and at selected times, respectively, t (min) is the time and k_2_ (µg/µg.min^−1^) is the rate constant of the pseudo-second-order adsorption. The intra-particle diffusion model was also used to find out the diffusion mechanism, denoted in Equation (7):

q_t_ = K_p_.t^1/2^ + C (7)

where q_t_ is the amount of protein adsorbed at the equilibrium (µg/µg) at time t, C (µg/µg) refers to the intra-particle diffusion constant related to the thickness of the boundary layer and K_p_ is the intra-particle diffusion rate constant K_P_ (µg/µg. min^1/2^) [50,51].

### 2.5. Thermodynamic Studies

GO (250 µg/mL) was dispersed in either BSA solution (900 µg/mL), collagen solution (315 µg/mL) or elastin solution (900 µg/mL). These mixtures were incubated for 2 h at different temperatures (5 °C, 10 °C, 15 °C, 25 °C, 37 °C and 39 °C). Samples were centrifuged for 10 min at 12,000 rpm and the supernatants were collected for the quantification of the non-adsorbed proteins with the BCA kit (Thermo Fisher). The three basic thermodynamic parameters—Gibbs free energy change (∆G°), entropy change (∆S°) and enthalpy change (∆H°)—were calculated using the following Equations (8)–(11) [29,52]:

Kd = qe/Ce(8)

∆G = −RT lnKd(9)

Ln Kd = −∆H/RT + ∆S/R(10)

∆G° = ∆H − T∆S (11)

where R is the gas constant (8.314 J/mol K), T is the absolute temperature (K), Kd is the equilibrium constant, q_e_ (µg/µg) is the amount of protein adsorbed per mass unit of GO at equilibrium and C_e_ (µg/mL) is the equilibrium concentration of the proteins. 

### 2.6. Electrochemical Study

First, sodium alginate solutions in combination with GO or GO-protein mixtures were prepared as follows: 1.87% (*w*/*v*) sodium alginate solutions were prepared in 1% mannitol and were then mixed with a GO suspension or mixtures of GO with the studied proteins at the aforementioned concentrations. With these reagents, the alginate hydrogels were elaborated. For this purpose, 2.7 mL of the previous solutions (alginate, alginate-GO and alginate-GO-proteins) were mixed with 60 μL of calcium sulphate 1.22 M and 240 μL of mannitol 1%, through two Luer Lock syringes (BS Syringe) connected with a Fluid Dispensing Connector (Braun), for 15 s. Then, the resulting mixtures were kept for gelification between two glass plates with a separation of 2 mm. The obtained hydrogels were cut into 14 mm diameter disks for electrochemical studies.

Electrochemical Impedance Spectroscopy (EIS) was conducted using a versa state-3 instrument (Princeton Applied research-USA), and a screen-printed electrode (Dropsens-Spain), based on carbon and a silver electrode for reference. The samples were immersed in 0.1 M PBS performing EIS measurements at room temperature, with a frequency range from 10^–1^ to 10^3^ Hz. Cyclic Voltammetry (CV) was performed to quantify the specific capacitance. The samples were immersed in 0.1 M PBS and CV measurements were carried out at the potential window of −0.5 to 0.2 V at various scan rates (100 mVs^−1^). The specific capacitance was calculated from the CV curves according to the following Equation (12) [53]:

C = Q/(2 Vm)(12)

where C (F·g^−1^) is the specific capacitance, Q (C) is the average charge during the charging and discharging process, V (Volt) is the potential window and m (g) is the mass of the hydrogel disk.

### 2.7. In Vitro Cell Viability Studies

The biological effects of alginate hydrogels containing GO or GO with different adsorbed proteins (BSA, collagen and elastin) were studied on murine C_2_C_12_ myoblasts genetically engineered to secrete erythropoietin (C_2_C_12_-EPO). The cells were grown in Dulbecco’s modified Eagle’s medium (Gibco) supplemented with 10% FBS, 2 mM L-glutamine and 1% antibiotic/antimycotic solution (basal medium) at 37 °C in a humidified atmosphere containing 5% CO_2_. The cells were passaged every 2–3 days. For the preparation of the C_2_C_12_-EPO containing hydrogels, the alginate, alginate-GO and alginate-GO-proteins (BSA, collagen or elastin) hydrogels were prepared as explained in Section 2.6 under aseptic conditions, filtering all of the solutions through a 0.20 μm syringe filter (Millipore, MA, USA). The myoblasts were harvested with 0.25% trypsin-EDTA, centrifuged and mixed with the hydrogels at a 5 × 10^6^ cells/mL cell density. Afterwards, the resulting mixtures were kept for gelling between two glass plates with 2 mm of thickness and disks were cut in aseptic conditions. The disks were cultured at 37 °C in a humidified atmosphere containing 5% CO_2_ with basal medium.

For fluorescence microscopy viability imaging, the hydrogels were stained with the LIVE/DEAD^®^ Viability/Cytotoxicity Kit (Invitrogen™) at different time points. The hydrogels were washed with DPBS and stained with 0.5 μM calcein AM and 0.5 μM ethidium homodimer-1. The samples were incubated at room temperature for 40 min, protected from light and observed under a Nikon TMS microscope (excitation/emission settings for calcein AM: 495/515 nm and for ethidium homodimer: 495/635 nm). At least three independent experiments were analyzed for each condition. For metabolic activity study, six disks from each condition were placed on 96-well plates, adding 100 μL of culture medium with 10 μL of Cell Counting Kit-8 solution (CCK-8, Sigma-Aldrich) per well. The plates were incubated inside a humidified chamber for 4 h at 37 °C. Then, the absorbance was read out on an Infinite M200 TECAN plate reader at 450 nm with a reference wavelength set at 650 nm. The secretion of EPO was also quantified from three disks/conditions. The disks were placed in 12-well plates adding 1 mL of the culture medium and collecting supernatants the next day. The amount of released EPO was quantified using the Quantikine IVD EPO ELISA kit (R&D Systems). All of the samples and standards were measured at least in triplicate. 

### 2.8. EPO and Insulin Adsorption Blocking Study

We evaluated the capacity of BSA, collagen and elastin for avoiding the adsorption of other proteins to the GO particle surface, such as EPO and insulin. First, GO-BSA, GO-collagen and GO-elastin mixtures were obtained as previously described in Section 2.5. These mixtures were incubated overnight with either recombinant EPO (200 mIU/mL) or insulin (150 mIU/mL). Next, the samples were spun for 5 min at 12,000 rpm, and then the supernatants were collected. The non-adsorbed EPO and insulin was quantified with the ELISA kits, the Quantikine IVD-EPO ELISA kit (R&D Systems) and the Insulin ELISA Kit (Mercodia), respectively. The GO without adsorbed proteins was considered as a control. All of the samples and standards were measured in duplicate. Three independent experiments were analyzed for each condition.

### 2.9. Statistical Analysis

Statistical analysis was done with SPSS software, version 24.00, or GraphPad Prism 8.0 (GraphPad Inc., San Diego, CA, USA). The data are shown as mean ± standard deviation. Values with *p* < 0.05 were considered significant for comparison between groups after confirming normality and performing ANOVA and Tukey’s post-hoc test for bivariate correlation. Pearson’s correlation coefficient was used for continuous data and Spearman’s for ordinal and nominal data.

## 3. Results and Discussion

In this experimental work, we studied how BSA, type I collagen and elastin, interact with the GO surface, analyzing their electrochemical characteristics after being embedded within alginate hydrogels. Next, we evaluated the biological impact of embedded C_2_C_12_-EPO cells within hybrid protein-coated GO particles with alginate hydrogels.

### 3.1. Raman Spectroscopy Shows the Functionalization of GO by BSA, Collagen and Elastin

**Figure 1:**
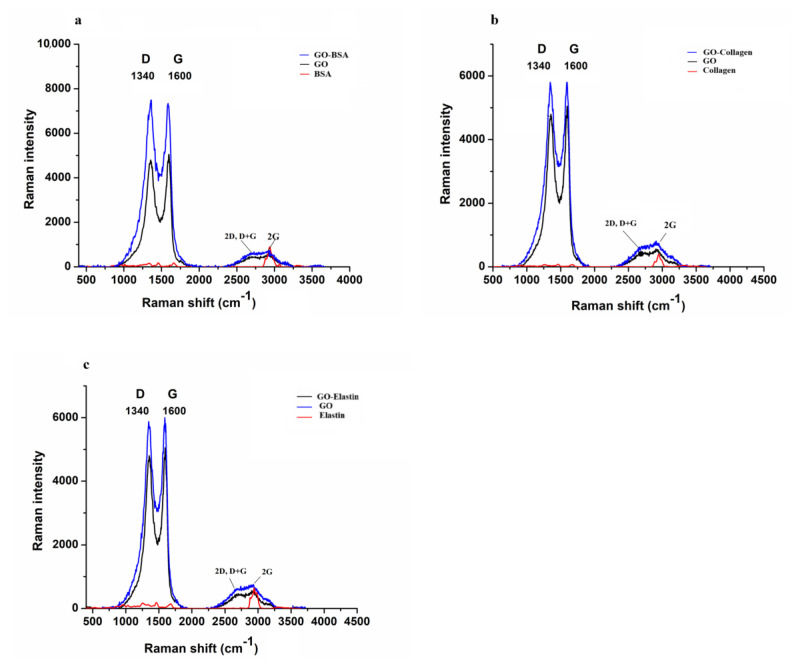
Raman spectrum of GO, BSA (**a**), collagen (**b**) and elastin (**c**), and the combination of each protein with GO.

The interactions between the studied proteins and GO were analyzed by Raman spectroscopy, obtaining the spectra of GO, BSA, collagen, elastin and the combinations of GO with each protein type, as shown in Figure 1. The proteins were hardly detected after mixing with the GO due to the higher Raman activity of the GO compared to the proteins. In the GO spectrum, two prominent peaks, commonly observed in sp^2^ graphite systems, corresponding to D (~1340 cm^−1^) and G (~1600 cm^−1^) bands, were clearly visible [54]. Moreover, the combination of 2D, D + G bands and 2G bands at 2500 cm^−1^ and 3200 cm^−1^ were detected, with a wide band around 3500 cm^−1^, maybe due to OH^-^ presence. More detailed analysis of the spectrum evidenced the presence of a band (I) at the low wavenumber side (1100–1250 cm^−1^) of the D band, usually attributed to sp^3^ bonds arising from broken sp^2^ rings or surface functionalization. 

Two components (G1 and G2) are required to fit the asymmetry of the G band. These excitations were present both in the GO and GO with protein spectra and were used to determine GO modifications after combining with protein. Detection at the same excitation wavelength (532 nm) did not show significant differences between the band wavenumbers of the GO and GO mixed with proteins, as shown in Table 1. Similarly, no appreciable wavenumber differences were observed among the three proteins mixed with the GO. However, interestingly, there was an evolution of the integrated band intensities comparing the GO and GO mixed with protein spectra. A constant D/G intensity ratio was observed for all of the proteins studied, within error, while the I/D ratio was enhanced after mixing the GO and proteins, shown in Table 1, indicating an increment in the functionalization of GO.

### 3.2. FTIR Spectroscopy Indicates a Formation of a Bio-Corona on the GO Surface

**Figure 2:**
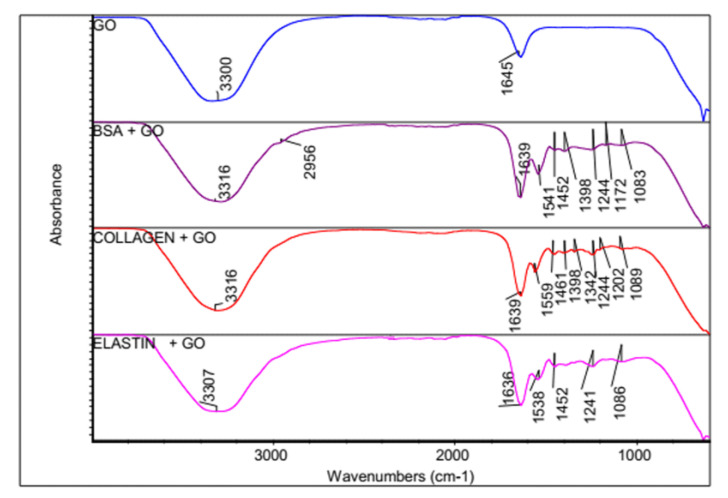
FTIR spectrum for GO, GO-BSA, GO-collagen and GO-elastin matrix.

In order to determine the adsorption of proteins on the GO surface, Fourier Transform Infrared Spectroscopy (FTIR) spectra from the GO of GO-proteins before and after the adsorption of the studied proteins was compared, as shown in Figure 2. The GO spectrum showed a broadband for H-bonded and an OH stretch at 3300 cm^−1^, a C=C characteristic band at 1645 cm^−1^ and a C-O stretch at 1056 cm^−1^ [54]. However, when the studied proteins were adsorbed on the GO surface, bands from the GO and proteins were overlapped. Thus, the FTIR spectrum for protein-coated GO showed NHCO stretching vibrations at 1636–1639 cm^−1^, characteristic of amide bonds from proteins and primary alcohol (C-OH stretch) at 1083–1089 cm^−1^. The amide II band from BSA was shifted from 1523 cm^−1^ to 1541 cm^−1^ when adsorbed within the GO, while collagen shifted from 1544 cm^−1^ to 1559 cm^−1^ and elastin from 1538 cm^−1^ to 1552 cm^−1^. Similarly, the amide III bands shifted from 1247 cm^−1^ to 1256 cm^−1^ with BSA, from 1250 cm^−1^ to 1244 cm^−1^ with collagen and from 1244 cm^−1^ to 1241 cm^−1^ with elastin. A peak at 1645 cm^−1^ in the GO spectrum, attributed to the aromatic C=C group of the sp^2^ carbon atom structure, was detected. However, this peak was not detected after the adsorption of the proteins, suggesting either the loss of the sp^2^ structure or the formation of a bio-corona on the GO surface. These results would indicate that the formation of such bio-corona on the GO surface would occur through the π–π interactions between the benzene ring from the proteins and the C=C from GO [55,56]. Nevertheless, other interactions, such as hydrogen bonds, could participate in the bio-corona formation.

### 3.3. Protein-GO Adsorption Capacity Is Related to the Protein Molecular Weight

**Figure 3:**
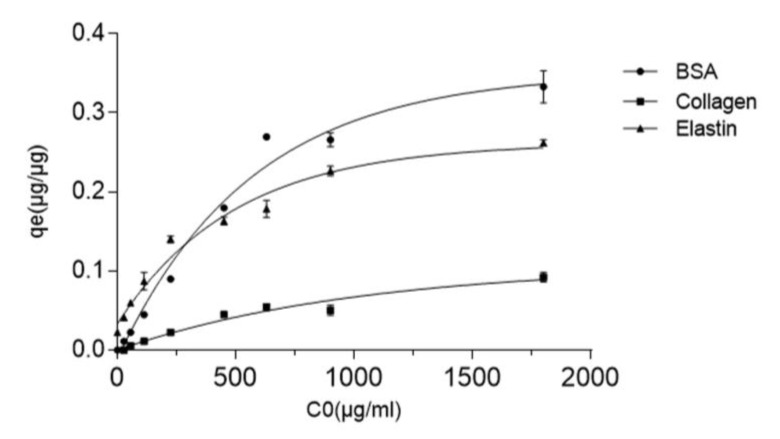
Effect on the GO adsorption capacity (qe) of the initial concentration (C_0_) of BSA, collagen and elastin at 37 °C after 2 h of incubation.

The adsorption capacity of GO platelets was studied by exposing them to different initial concentrations of BSA, collagen and elastin. From the quantified data, it was clear that the adsorption capacity of GO increased when the initial concentration of the proteins was enhanced, as shown in Figure 3. In fact, an increment in the initial protein concentration accelerated the diffusion of more protein from the solution towards the GO particle surface, indicating that the initial adsorbed protein provides the needed driving force for overcoming the resistance to the mass transfer of the protein between the aqueous phase and the GO particles’ solid phase [57]. However, this effect seems to be different among the three studied proteins. Thus, BSA-GO was the most affected interaction by the increment of the initial protein concentration, showing a q_e_ of 0.045 ug/ug at an initial BSA concentration of 112.5 µg/mL and a 5.9-times increment at an initial BSA concentration of up to 1000 µg/mL. The maximum BSA adsorption capacity value on the GO surface was 0.332 ± 0.02 µg/µg. In contrast, collagen was the least affected in terms of GO surface adsorption when modifying the initial concentration, with the highest qe value at 0.092 ± 0.005 µg/µg.

All of the studied protein-GO interactions reached a plateau, indicating that all of the active sites of the GO surface were occupied. Since BSA and elastin are small molecular weight proteins, with 66.5 KDa and 70 KDa, respectively, while collagen has 300 KDa, we consider that the theoretical Random Sequential Adsorption (RSA) model can explain this adsorption process. In the RSA model, adsorption is described as a stochastic process with particles successively placed onto a surface where other particles have already existed. According to the RSA model, proteins will be adsorbed if they are not overlapped with previously adsorbed proteins (steric repulsion) [58]. Based on this model, collagen would show higher steric repulsion than BSA or elastin, because higher molecular weight would have more chance of overlapping with other collagen molecules on the GO surface, reflecting in a lower adsorption capacity than BSA or collagen, as observed.

### 3.4. Proteins Are Adsorbed in GO Platelets as a Monolayer

Langmuir and Freundlich models were applied to the adsorption capacity experimental data at constant temperature to determine the adsorption performance on the GO surface from the studied proteins, as shown in Table 2. Experimental data from the three proteins fitted to Langmuir model with a R^2^ value between 0.97–0.99, suggesting that the adsorption process occurs on the homogeneous surface. Moreover, the calculated q_max_ values from the three proteins are close to the q_e_ experimental results, indicating that this model describes the adsorption of the proteins in contact with the GO particles in suspension. Therefore, we can conclude that the three proteins cover the finite number of adsorption sites from the surface of the GO platelets as a monolayer, without transmigration along the plane of the surface [51] and without interactions between the adsorbed proteins along the surface [59]. Since, no good R^2^ values were detected after applying the Freundlich model, we can discard the adsorption of an heterogeneous adsorbent with the formation of multiple layers of adsorbed proteins [52,60]. Separation factor values (R_L_) below 1 indicated a favorable adsorption into the GO surface of the three studied proteins, with the indication of an interaction with BSA with a value close to zero [51].

### 3.5. Kinetic Study of the Protein Adsorption into GO Shows that Lower Molecular Weight Proteins Are Adsorbed Faster

**Figure 4:**
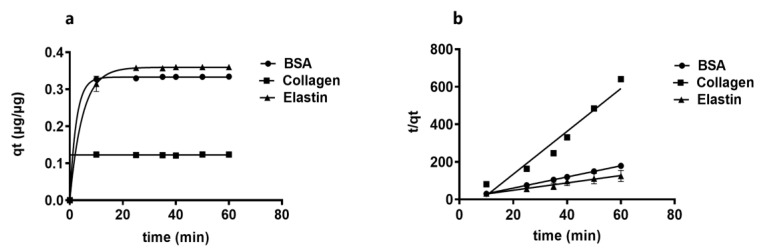
Kinetic study of protein adsorption and the GO adsorption capacity for BSA, collagen and elastin: (**a**) Representation of adsorption capacity (q_t_) over time. (**b**) Pseudo-second-order kinetic model.

Next, we studied the kinetics of the protein adsorption, detecting that the rate of adsorption rapidly increases after proteins and GO platelets are mixed, due to the high available number of active sites on the GO platelets surface, shown in Figure 4a. The GO demonstrated a high capacity to adsorb the studied proteins on its surface and, ten minutes later when equilibrium state was reached, adsorption gradually slowed down since fewer sorption sites were available.

To understand the nature of this process, both pseudo-first-order and pseudo-second-order kinetic models were applied. The experimental data did not fit on the pseudo-first order kinetic mode, indicating that adsorption does not occur between one protein and one sorption site on the GO solid surface. However, the data did fit onto the pseudo-second-order kinetic model, shown in Figure 4b and Table 3, suggesting that each protein can be adsorbed into two sorption sites on the GO platelets [56]. Among the three proteins, collagen showed the lowest affinity based on its q_t_ value, maybe due to its higher molecular weight (300 kDa), compared to BSA (66.5 kDa) and elastin (70 kDa) which increase steric repulsion forces, shown in Figure 4.

**Figure 5:**
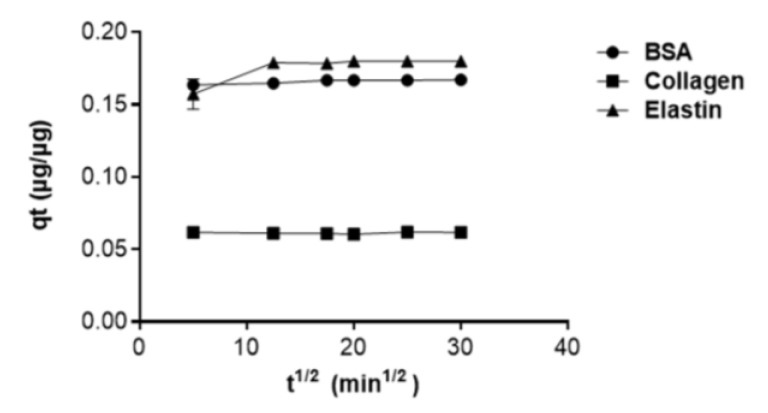
Intra-particle diffusion model plot.

Next, to find the mechanism that fits the uptake of the protein into the GO surface, we applied the intra-particle diffusion model, a common study for material adsorption on solid adsorbents, such as GO, which would assume the adsorption process in three steps: the diffusion of molecules from the bulk solution to the external surface of GO particles; a diffusion through the internal surface of GO pores; a final adsorption into the internal sites of GO [44]. According to this model, elastin and BSA showed higher adsorption capacities (q_t_) than collagen (I). However, the plot of q_t_ against t^1/2^ was linear, as shown in Figure 5, indicating that the intra-particle diffusion is not the unique process interposed in the GO adsorption of proteins, but film diffusion is also involved [50,61].

Values calculated from the intra-particle diffusion model indicate that elastin and BSA have higher Kp (as the intra-particle diffusion rate is constant), than collagen, which is similar to C, the thickness of the boundary layer, shown in Table 4. We consider that Kp and C provide an indication of the high affinity of BSA and elastin to the GO surface through the hydrophilic–hydrophilic interactions of the proteins to the carbon material. In contrast, interfering forces from the hydrophobic interaction with collagen, in addition to its high molecular weight, would be responsible of the lower Kp and C values.

### 3.6. Protein Molecules Determine Thermodynamic Behavior in Their Adsorption by GO

**Figure 6:**
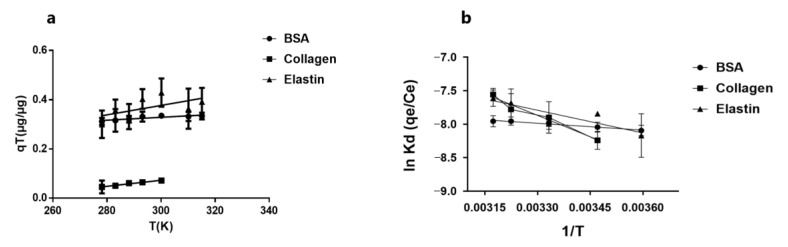
(**a**) Effect of temperature on adsorption capacity of BSA, collagen and elastin onto GO platelets surface. (**b**) van ’t Hoff plot: K_d_: equilibrium constant; T: absolute temperature in K.

We studied the effect of temperature on the protein adsorption into the GO platelets surface, since a change in temperature could modify the protein adsorption into GO. Therefore, we analyzed the protein adsorption from 278 K to 315 K, and calculated the parameters ∆H°, ∆S° and ∆G° with Equations (8)–(11), shown in Table 5. We detected an increase in adsorption capacity with the increment of temperature, as shown in Figure 6. The positive ∆H° values indicate that the adsorption of proteins on the GO surface is endothermic, evidencing that the interaction of BSA with GO is weaker than collagen and elastin, as observed with its lower ∆H° value [62]. Since adsorption is the sum of two steps, the endothermic hydration of the protein in the solution and the exothermic adsorption on the GO surface [53], the positive ∆H° values indicate that, in the protein adsorption on GO, hydration is the most predominant step.

Entropy at this range of temperature for the three studied proteins was negative (ΔS° < 0), indicating a decrease in randomness after the adsorption of the proteins into GO, in accordance with common protein behavior [1,51,60]. The highest ∆S° was detected in collagen while the lowest was in BSA, suggesting again that protein molecular weight plays an important role. Finally, the positive ∆G° values, characteristic of an endergonic reaction, suggest a non-spontaneous adsorption process of these proteins and an energy barrier for proteins to diffuse from the solution to the GO surface, with a hydration shell around the proteins that could prevent its adsorption into GO. Moreover, the values below 40 KJ/mol indicated not a physisorption process [43,54,56], but a chemisorption process instead.

### 3.7. Conductivity Is Improved after Coating GO with Proteins within Alginate Matrixes

**Figure 7:**
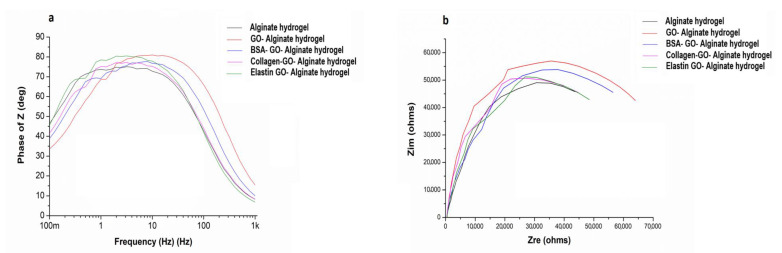
Electrochemical study from protein coated-GO alginate hydrogels compared to alginate hydrogels. (**a**) Bode plots, (phase angle Z vs. frequency from 10^−1^ to 10^3^ Hz. (**b**) Nyquist diagram, Zim vs. Zre (ohm).

GO is an electrically insulating material due to its disrupted sp^2^ bonding networks, but it can act as a semiconductor, depending on the degree of oxidation [63], mediating as an electrochemical mediator in contact with cells. Therefore, we studied the electrochemical activity of protein coated-GO-hydrogels. We first determined the electrochemical impedance spectroscopy by measuring the phase impedance (Z) in the frequency range of 10^−1^–10^3^ Hz, as shown in Figure 7. The data indicated an insulating/conducting behavior in the studied hydrogels.

We quantified the phase angles of Z from all the samples and represented them in Bode plots, shown in Figure 7a. Although the data at the lowest frequencies could not be represented due to the noise caused by the high values of impedance, we could detect that the phase angle tended to decrease towards zero at the high-frequencies region, reaching close to 90 degrees at lower high-frequencies, shown in Figure 7a. However slight modifications could be detected when GO or protein-coated GO was embedded in the alginate matrixes. We also quantified the impedances (Z_im_) from the different samples and represented them in Nyquist plots, shown in Figure 7b, detecting slight differences after embedding GO or coated-GO within alginate hydrogels. GO-alginate matrixes showed higher impedance than alginate hydrogels. However, although the presence of GO increased the impedance, protein-coating the GO decreased it, indicating an improvement in conductive behavior. The disrupted sp^2^ bonding networks from GO provided an insulating behavior [63], but coating with proteins could have led to the recovery of the sp^2^ networks, improving its conductivity properties [64], and therefore, creating a suitable signal conduction between cells and the alginate matrix when used in vivo. In fact, conductivity, among other GO-containing scaffold properties, would help to overcome the limitations from metals and silicon implantable devices, providing safer and effective treatments for pathological conditions in the clinic, particularly in the field of neurology or cardiology [65,66,67].

### 3.8. Capacitance Is Reduced after GO Protein Coating

**Figure 8:**
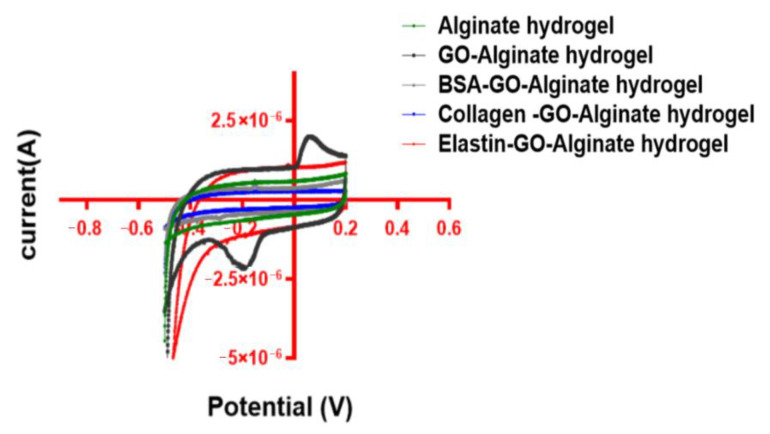
Voltammograms from protein-coated GO alginate and alginate hydrogels.

We determined the capacitance (Z_im_) of the protein-coated alginate hydrogels by cyclic voltammetry (CV) at scan rates of ± 100 mV, in a potential window of −0.5 to 0.2 V, as shown in Figure 8.

The calculated non-coated GO-alginate hydrogel capacitance was the highest among the studied hydrogels (2.78 × 10^3^ f/g), 5.64-times higher than alginate hydrogels (4.77 × 10^4^ f/g). However, we detected a reduction in the capacitance of the protein-coated GO-alginate hybrid hydrogels compared to GO-alginate hydrogels. BSA-GO and collagen-GO containing hydrogels showed a value of 7.04 × 10^4^ f/g and 4.98 × 10^4^ f/g, smaller than the hydrogels containing elastin (2.47 × 10^3^ f/g). This reduction in the GO capacitance after protein coating is in agreement with those observed in EIS measurements, indicating that there is an accelerated electron transfer evidenced by a decreased Z_im_, lower phase shift and smaller impedance after the adsorption of proteins by GO sheets [68]. We consider that impedance decrease, in combination with observed capacitance measurements, indicate a slight improvement in the conductivity of protein-coated GO hybrid alginate hydrogels.

### 3.9. Collagen and Elastin Coated GO Improves Alginate Hydrogel-Embedded C_2_C_12_ Cell Viability

**Figure 9:**
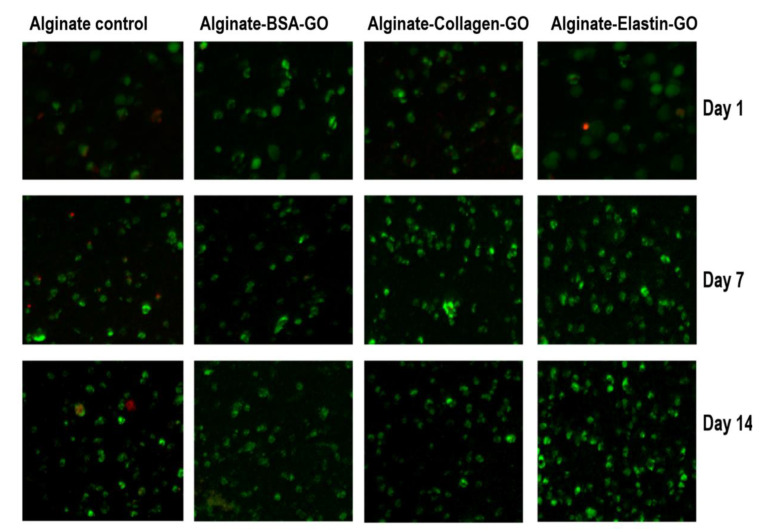
Fluorescence microscopy images, after calcein/ethidium staining for C_2_C_12_–EPO myoblasts incorporated within the modified alginate hydrogels based on GO-BSA, GO-collagen and GO-elastin matrices. Green: live cells. Red: dead cells. Scale bar: 100 µm.

The introduction of FBS-coated GO nanoparticles in the matrix of alginate-poly-l-lysine-alginate hydrogels has shown to enhance the viability of the encapsulated erythropoietin-releasing C_2_C_12_ myoblasts (C_2_C_12_-EPO) [10,47,48], but if a sole protein could reproduce, this enhancement has not been discerned yet. Therefore, after characterizing the interaction between BSA, collagen or elastin with the GO surface, we generated alginate-based hydrogels containing protein-coated GO platelets to embed C_2_C_12_-EPO cells in order to study the in vitro outcomes from each protein-coated GO on a 3D model. Cell viability, assessed by confocal microscopy, showed that one day after embedding the cells, there were no differences among all the studied groups of hydrogels, with a similar number of live and dead cells, shown in Figure 9. However, one week later, there was a noteworthy cell viability enhancement from hydrogels containing collagen- and elastin-coated GO particles, increasing even more the second week in the elastin-coated GO group, indicating that collagen, and especially elastin, are able to improve the viability of embedded C_2_C_12_ cells within alginate hydrogels.

**Figure 10:**
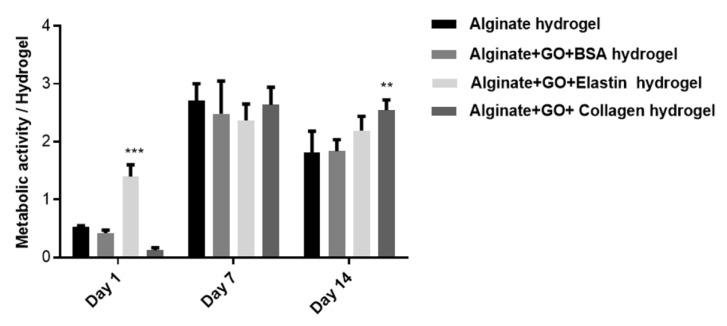
Metabolic activity of hybrid alginate-GO embedded C_2_C_12_-EPO myoblasts over two weeks. Note: **: *p* < 0.01; ***: *p* < 0.001 compared with cells encapsulated in alginate without GO.

Next, we complemented the imaging studies through the quantification of metabolic activity. On the first day, the metabolic activity of the embedded cells was low with all of the studied protein-coated GO, perhaps due to the high shear stress that cells suffer during hydrogel fabrication after 24 h, shown in Figure 10. However, a significantly higher metabolic activity (*p* < 0.001) was already detected at this time point from the elastin-coated GO hydrogels. One and two weeks later, metabolic activity had increased over all of the hydrogels studied, showing only a statistically significant increment (*p* < 0.01) at two weeks in collagen-coated GO samples, as shown in Figure 10.

### 3.10. Protein Release by Embedded Cells Is Influenced by the Type of Protein-Coated GO

**Figure 11:**
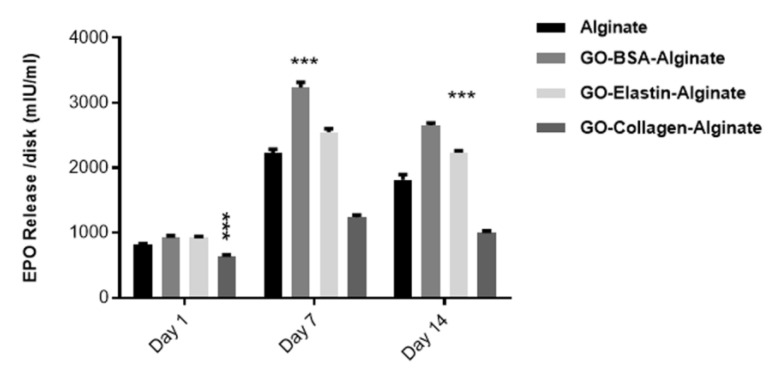
EPO production and release from C_2_C_12_-EPO myoblasts embedded in different hybrid protein-GO-alginate hydrogels. Note:; ***: *p* < 0.001 compared with cells embedded in alginate without GO.

Next we quantified if the different protein-coated GO platelets had any effect on the production and release of the therapeutic protein, EPO. The BSA-coated GO containing alginate hydrogel showed the highest EPO release among the analyzed groups, while the elastin group had a similar profile in comparison to the control and the collagen group released a lower amount, as shown in Figure 11. These results were difficult to foresee, taking into account the viability results obtained with the calcein/ethidium staining, shown in Figure 9. We expected that a higher cell viability would represent a higher release of the therapeutic protein EPO. In addition, the collagen-GO-alginate hydrogels did not show lower viability in comparison to the controls but released a lower amount of EPO. One possible explanation is that elastin and collagen proteins are not able to form a stable and uniform bio-corona around the GO platelets of the release protein before their use for hydrogel fabrication, therefore avoiding the GO-inherent adsorption of the released proteins. Thus, although cells could be producing high quantities of EPO, the therapeutic protein could be retained in the GO surface, avoiding its release.

**Figure 12:**
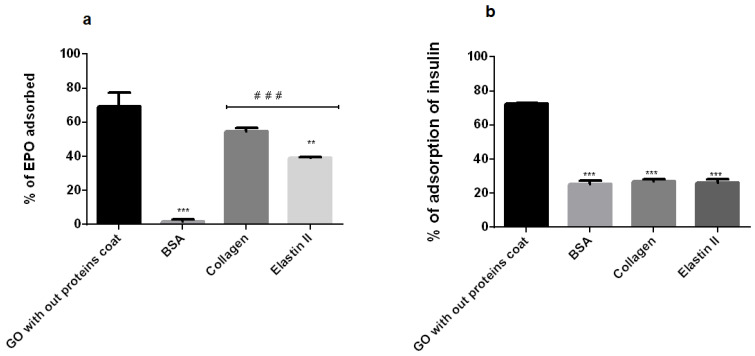
Percentage of (**a**) EPO and (**b**) insulin adsorbed by GO without coating, and BSA-, collagen- and elastin-coating. Incubation conditions: GO final concentration, 50 µg/mL; temperature, 37 °C; incubation time, 24 h. Note: *p* < 0.01; **: *p* < 0.001; *** compared with non-coated GO. ###: *p* < 0.001 compared to BSA coated-GO.

In order to confirm this hypothesis, we studied the capacity of BSA, elastin and collagen to block the GO platelets surface and avoid the adherence or interaction of EPO, shown in Figure 12a. Thus, the GO platelets were able to adsorb the 70% of the recombinant protein EPO when mixed and incubated in vitro. Interestingly, the BSA pre-coating of the GO nanoparticles was able to reduce this adsorption almost completely, which means that all of the EPO produced by the encapsulated C_2_C_12_ myoblast inside the BSA-GO-alginate hydrogels should be released into the culture media. In contrast, collagen and elastin reduced the percentage of EPO adsorption, but not completely, suggesting that some of the therapeutic protein can be retained within the hydrogels adsorbed into the GO particles. These results would explain why the amount of therapeutic protein detected on the culture media was similar to the alginate control group, although higher levels of viability were detected from elastin-GO-alginate hybrid hydrogels. Thus, although cells in the elastin group would produce higher amounts of protein (39.71% more than the detected levels), we were not able to quantify this difference. Regarding collagen, it was only able to block 45.29% of the EPO protein, indicating that 54.71% of the produced EPO should be retained within the hydrogels, shown in Figure 12a. However, even if we take into account this low blocking capacity, the amount of protein released by the cells on this hybrid hydrogel would be lower than in the BSA or elastin group. Therefore, we believe that another mechanism could be affecting the low protein release in collagen-coated GO hydrogels, such as the adsorption of EPO not only by the GO platelets, but also by collagen itself, since EPO has a tendency to cross-link soluble type IV collagen in vitro [69,70].

Aiming to establish if the blocking effect of the assayed three proteins has the same effect on different therapeutic proteins, we performed this study with insulin, since insulin producing cells in alginate microcapsules or hydrogels are a wide field of investigation for the treatment of type one diabetes mellitus [71,72]. Insulin showed a similar percentage of the adsorption of EPO to the GO surface, close to 70%. However, in contrast to EPO, the three studied proteins had the same blocking capacity in the interaction between the GO particles and insulin, reducing the adsorption of insulin into the GO surface by up to 60%, shown in Figure 12b. Therefore, we hypothesized that the hydrophilic nature of BSA and elastin interferes with the hydrophobic nature of EPO [73], decreasing the affinity of EPO to be adsorbed on the GO surface, in contrast to the hydrophobic nature of collagen, which would interact with the therapeutic protein, reducing its ability to prevent the trapping of EPO on the GO surface. Regarding insulin, its small molecular weight (6 kD) and its high hydrophilic nature, could possibly compete with the studied proteins at the GO binding sites, replacing part of the coating protein, or being inserted into the void spaces between the adsorbed proteins.

## 4. Conclusions

We have confirmed the formation of protein layers on the GO surface by Raman spectroscopy and FTIR, studying BSA, collagen and elastin. To understand the mechanisms underlying the bio-corona formation, we have described how the protein–GO adsorption capacity is related to the protein molecular weight, showing that collagen, with higher molecular weight, would have higher steric repulsion by overlapping with other collagen molecules on the GO surface. Moreover, we have concluded that proteins are adsorbed in the GO platelets as a monolayer, since the three proteins fit into the Langmuir model, with faster adsorption in lower molecular weight proteins. In this adsorption, each protein would be adsorbed by two sorption sites on the GO platelets, fitting to the pseudo-second-order models and following an endothermic process. The functionalization of GO with the studied proteins would alter the electrochemical activity of GO, decreasing its impedance and its specific capacitance, which could improve the biocompatibility of the carbon platelets.

All these mechanisms would be reflected in better cell viability and EPO release in hydrogels based more on GO-BSA and GO-elastin, than on GO-collagen. However, the hydrophilic nature of the adsorbed protein would play an important role in the biocompatibility of protein-coated GO, perhaps by attracting cells via π–π interactions and boosting more cells to adhere and proliferate on these matrices. Finally, although cells could be producing high quantities of EPO within the hybrid hydrogels, the therapeutic protein is retained in the GO surface, preventing its release. This retention is dramatically observed in the collagen-coated GO hybrid hydrogels, precluding their clinical translation. Therefore, we conclude that BSA- or elastin-coated GO hybrid hydrogels could act as promising scaffolds for improving the viability and functionality of embedded cells.

## Figures and Tables

**Table 1:** Raman spectroscopy data from the GO and protein-coated GO nanoparticles (proteins = BSA, collagen or elastin).

Wavenumbers (cm^−1^) at 532 nm Excitation Wavelength	GO	GO + Protein
(I)	1245	1230–1250
(D)	1354	1352–1354
(G1)	1569	1530–1570
(G2)	1603	1595–1605
**Band Intensity Ratio**	**GO**	**GO + Protein**
I/D	0.25	0.45–0.48
D/(G1 + G2)	1.3	1.1–1.5
(D + I)/(G1 + G2)	1.7	1.6–2.2

**Table 2:** Parameters calculated from experimental data for Langmuir and Freundlich models. Notes: q_e_: amount of protein adsorbed per GO weight at equilibrium; qmax: maximum amount of protein adsorbed per GO weight; K_L_: Langmuir constant; R_L_: separation factor; n: adsorption intensity; K_F_: Freundlich constant.

	Langmuir Model					Freundlich Model			
	q_e_ (µg/µg)	q_max_ (µg/µg)	K_L_ (mL/µg)	R_L_	R^2^	1/n	N	K_F_	R^2^
BSA	0.332	0.330	0.057	0.009	0.99	0.050	22.030	0.225	0.750
Elastin	0.262	0.380	0.002	0.249	0.97	0.380	2.600	0.014	0.950
Collagen	0.122	0.071	0.023	0.044	0.97	0.200	4.780	0.019	0.700

**Table 3:** Parameters calculated from the pseudo-second-order kinetic model.

	q_e_ (µg/µg)	K_2_(µg/µg.min)	R^2^	q_t_ (µg/µg)
BSA	0.336	7.220	0.990	0.332
Collagen	0.362	12.860	0.990	0.123
Elastin	0.125	4.158	0.990	0.352

**Table 4:** Parameters calculated from intra-particle diffusion model. Note: Kp, intra-particle diffusion rate constant; C, the thickness of the boundary layer.

	Kp (µg/µg.min^1/2^)	C	R^2^
BSA	0.00014	0.163	0.91
Collagen	1.15 × 10^−5^	0.060	0.37
Elastin	0.00078	0.161	0.70

**Table 5:** Thermodynamic parameters. Enthalpy change: ΔH°; Entropy change: ΔS°; Gibbs free energy change: ΔG° at 300 K.

	ΔH° (kJ/mol)	ΔS° (kJ/mol.K)	ΔG° (kJ/mol)	R^2^
BSA	2.598	−0.057887	20.543	0.981
Elastin	16.270	−0.008881	19.031	0.779
Collagen	17.363	−0.006935	19.513	0.935
